# Screening key genes for intracranial aneurysm rupture using LASSO regression and the SVM-RFE algorithm

**DOI:** 10.3389/fmed.2024.1487224

**Published:** 2025-01-06

**Authors:** Qi Wu, Chunli Yang, Cuilan Huang, Zhiying Lin

**Affiliations:** Jiangxi Provincial People's Hospital, The First Affiliated Hospital of Nanchang Medical College, Nanchang, China

**Keywords:** LASSO regression, SVM-RFE algorithm, intracranial aneurysm, ROC curve, PPI network

## Abstract

**Background:**

Although an intracranial aneurysm (IA) is widespread and fatal, few drugs can be used to prevent its rupture. This study explored the molecular mechanism and potential targets of IA rupture through bioinformatics methods.

**Methods:**

The gene expression matrices of GSE13353, GSE122897, and GSE15629 were downloaded. Differentially expressed genes (DEGs) were screened using the limma package. Functional enrichment analysis was performed, and a PPI network was constructed. Furthermore, candidate key genes were identified using the least absolute shrinkage and selection operator (LASSO) regression model, support vector machine-recursive feature elimination (SVM-RFE) analysis, and PPI network analysis. ROC analysis was conducted to further verify the diagnostic value of the key genes.

**Results:**

A total of 334 DEGs were screened, including 175 upregulated genes and 159 downregulated genes. Further functional analysis suggested that the DEGs were enriched in inflammation and immune response pathways. Fourteen hub genes were identified using the two algorithms. The PPI networks of the hub genes were analyzed using the Cytoscape plugin CytoNCA to obtain two key genes (IL10 and Integrin α5 (ITGA5)). The ROC curve analysis showed that the AUC values of IL10 and ITGA5 were 0.801, and 0.786, respectively. In addition, the two key genes were significantly positively correlated with macrophages and Treg (T) cells. The immune score and ESTIMATE score of the ruptured IA group were significantly higher than those of the unruptured IA group.

**Conclusion:**

The increase in IL-10 and ITGA5 may weaken the vascular wall by promoting inflammation in blood vessels and immune cells, which could have a harmful effect on the rupture of IAs.

## Introduction

An intracranial aneurysm (IA) is an irreversible dilation of the intracranial arterial wall (1). Unruptured IAs account for 3% of the general population, with rupture and bleeding being the main risks (2). Currently, the surgical treatments for IAs are endovascular coiling and surgical clipping (3, 4), both of which can lead to various complications. Except for these two invasive procedures, there are no available preventive treatments for IAs prior to rupture (5). Thus, the study aimed to explore the molecular mechanisms of IA rupture and identify key genes to prevent IA rupture. This could help improve the treatment strategy for this devastating complication.

Inflammation, immune response, and hemodynamic stress play important roles in the occurrence, development, and rupture of IAs (6–8). Recently, Tutino VM indicated that the expression of IA-related mRNA in peripheral blood neutrophils of patients with aneurysms was increased (9). IAs are closely related to several inflammatory factors, such as IL-1β and IL-6 (10, 11). Pera et al. compared ruptured IAs with unruptured IAs and a normal artery group, respectively, and found that the differentially expressed genes (DEGs) were enriched in the immune response (12).

In our study, we made a preliminary attempt to elucidate biomarkers and the molecular mechanisms of IA rupture. We downloaded the gene expression matrices of GSE13353, GSE15629, and GSE122897. We further identified differentially expressed genes based on the gene expression matrices. Subsequently, function enrichment analyses (including GO analysis, KEGG enrichment pathway analysis, and Disease Ontology (DO) enrichment analysis) of the DEGs were carried out. Fourteen genes were screened using least absolute shrinkage and selection operator (LASSO) and support vector machine-recursive feature elimination (SVM-RFE) analysis. Furthermore, we built a PPI network of the fourteen genes to further identify the key genes. Finally, ROC curve analysis was used to verify the selected key genes.

## Materials and methods

### Data collection

The datasets GSE13353, GSE15629, and GSE122897 were selected from the Gene Expression Omnibus (GEO) database. Among them, GSE13353 included eight cases of unruptured IAs and 11 cases of ruptured IAs, GSE15629 included six cases of unruptured IAs and eight cases of ruptured IAs, and GSE122897 included 21 cases of unruptured IAs and 21 cases of ruptured IAs.

### Differentially expressed genes (DEG) screening

We obtained the gene expression matrices and merged them into one matrix. Then, the processed gene matrix files were run in the impulse package and the limma package of R software, and missing value estimation and logarithmic conversion were performed on the data to obtain normalized data.

### Functional enrichment analysis

Go and KEGG pathway analyses were carried out on the DEGs in each module to better understand their biological functions. According to the results of the functional analysis, the differences between the ruptured IA and unruptured IA groups were compared. DO enrichment analysis is a method used to identify and understand disease-related gene sets in biomedical research. It uses DO to organize and analyze data, which is helpful to discover the potential relationship between genes and diseases. DO is a tool for annotating genes from the perspective of diseases, which is very important for translating high-throughput sequencing results into clinical applications. DO provides a framework for integrating disease-related information and object annotations and supports the exploration of functional similarities between diseases and genes. It plays an important role in the organization, representation, and standardization of disease knowledge. DO can be used to guide diagnosis and the development of disease phenotypes and drug association prediction.

### Model building

To predict the IA status, we used two algorithms to select sepsis characteristic genes. LASSO regression is a linear model that compresses the model coefficients by adding a penalty term, performs variable selection, and reduces model complexity. This penalty term is a function of the sum of the absolute values, which compresses some coefficients to zero, thus achieving the purpose of variable selection. In LASSO regression analysis, the strictness of variable selection can be controlled by adjusting the coefficient *λ* of the penalty term. We built a LASSO regression model using the “glmnet” package to prevent over-fitting, and the optimal value of penalty parameter *λ* was determined by 10-fold cross-validation. Therefore, we could increase the plasticity of selecting hub genes (13–15).

An SVM is a supervised machine learning technology for disease classification (16). The SVM algorithm focuses on classification tasks and achieves the separation of different types of samples by finding the optimal combination, but it does not have the ability for feature selection. The RFE algorithm iteratively eliminates unimportant features so that feature selection can be carried out simultaneously with model training and the feature subset that has the highest influence on the classification results can be selected. In each iteration, the weights of features are recalculated, which can allow for better consideration of the correlation between features and help avoid the loss of information during the feature selection process. The method for SVM-based hub gene screening primarily involves RFE combined with an SVM, known as SVM-RFE. By eliminating unimportant features, the SVM-RFE algorithm can reduce the dimension of feature space and the complexity of the model, thus improving the generalization ability of the model. The selected feature subsets are often more interpretable because they are the most representative feature combinations in the data. In addition, the algorithm automatically calculates feature importance and iterates, thus reducing the possibility of artificially selecting variables. An SVM correctly classifies any linearly separable data and then fully separates these classified data. SVM-RFE selects the most important genes based on the weights assigned by the classifier (17).

### Construction and module analysis of the PPI network

Overlapping genes are obtained by intersecting the genes identified by the LASSO regression model and SVM-RFE, which are then used for further PPI network analysis. The PPI network is then imported into Cytoscape software (18). The CytoNCA plugin is used for centrality analysis, which includes three parameters: degree, betweenness, and eigenvector (19). The top 20% of nodes for each parameter are considered important nodes in the CytoNCA analysis, and the genes represented are regarded as key genes in centrality analysis.

### Immunoinfiltration analysis

ssGSEA was performed to explore the differences in infiltration levels within the IA expression profile using the R package GSVA. The ESTIMATE algorithm evaluates the tumor microenvironment (TME) based on the immune score, stromal score, and ESTIMATE score.

## Results

### Screening for DEGs

**Figure 1:**
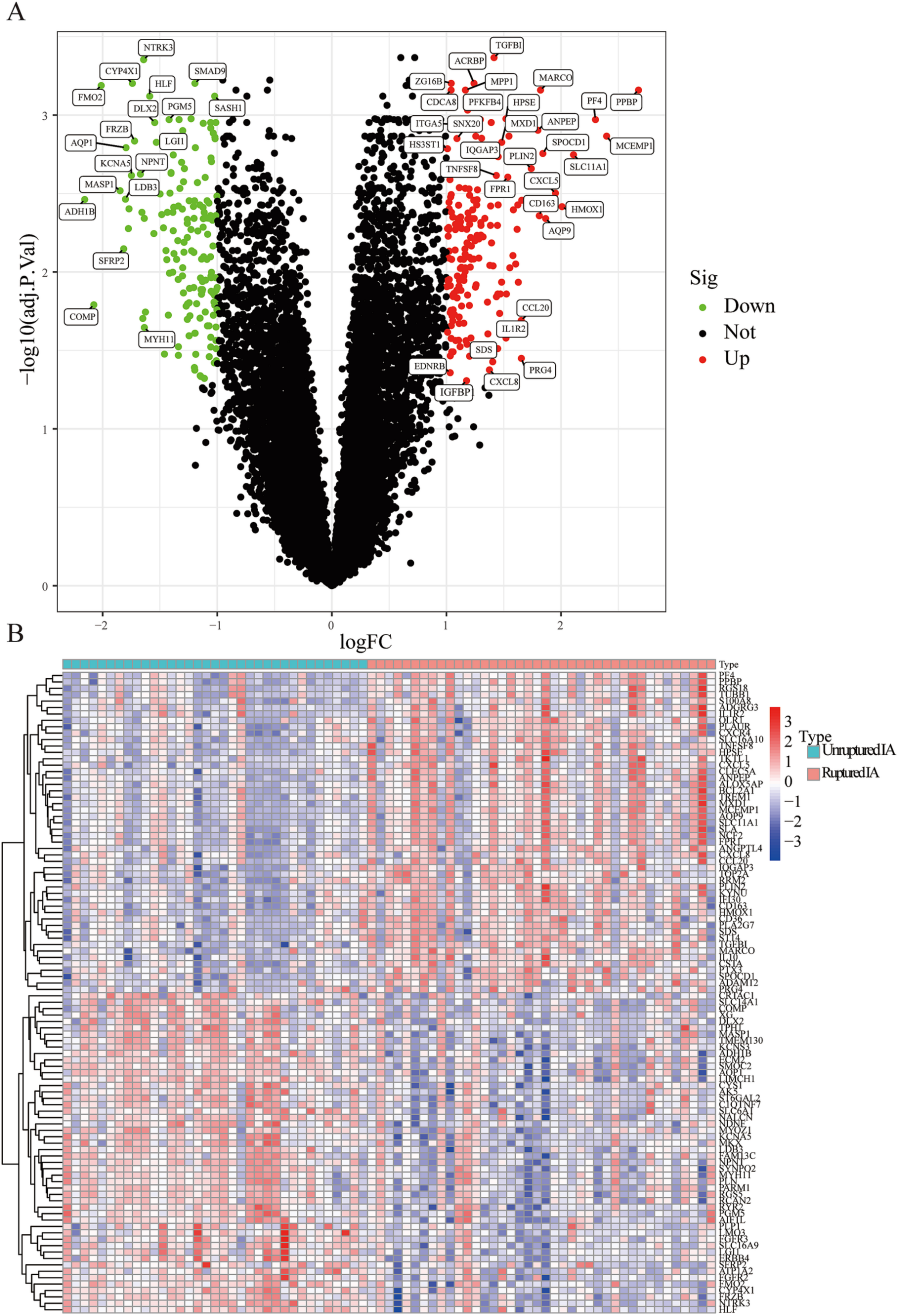
The screened DEGs in IA from the GSE13353, GSE122897, and GSE15629 datasets. **(A)** Volcano plot visualizing the DEGs identified using the limma package. The red and green points represent the significantly upregulated and downregulated DEGs, respectively; **(B)** Heatmap visualizing the top 20 upregulated and downregulated DEGs.

A total of 334 DEGs were identified from the integration matrix. The DEGs comprised 175 upregulated genes and 159 downregulated genes, based on the criteria of |log_2_(FC)| > 1 (Figure 1A). The top five upregulated genes were PPBP, MCEMP1, PF4, SLC11A1, and HMOX1. The top five down-regulated genes were ADH1B, COMP, FMO2, MASP1, and SFRP2, respectively (Figure 1B; Table 1). The PPI network built from the DEGs consisted of 334 nodes and 13,438 edges (Supplementary Figure 1).

**Table 1:** The 10 upregulated and downregulated genes among the DEGs.

Gene	log2 (fold change)	*p*
CD163	1.810304	0.000105
MARCO	1.820724	1.07E-06
SPOCD1	1.840896	1.46E-05
AQP9	1.865355	0.000118
CXCL5	1.950924	5.03E-05
HMOX1	2.007942	7.67E-05
SLC11A1	2.108484	1.50E-05
PF4	2.29994	2.86E-06
MCEMP1	2.397431	7.60E-06
PPBP	2.675522	9.65E-07
ADH1B	−2.15643	5.90E-05
COMP	−2.07683	0.001197
FMO2	−2.01231	7.52E-07
MASP1	−1.84773	4.54E-05
SFRP2	−1.81636	0.000276
LDB3	−1.80072	5.86E-05
AQP1	−1.79598	1.25E-05
ATP1A2	−1.77504	0.000163
TMEM130	−1.76982	8.10E-05
KCNA5	−1.74819	2.68E-05

### Functional analysis

**Figure 2:**
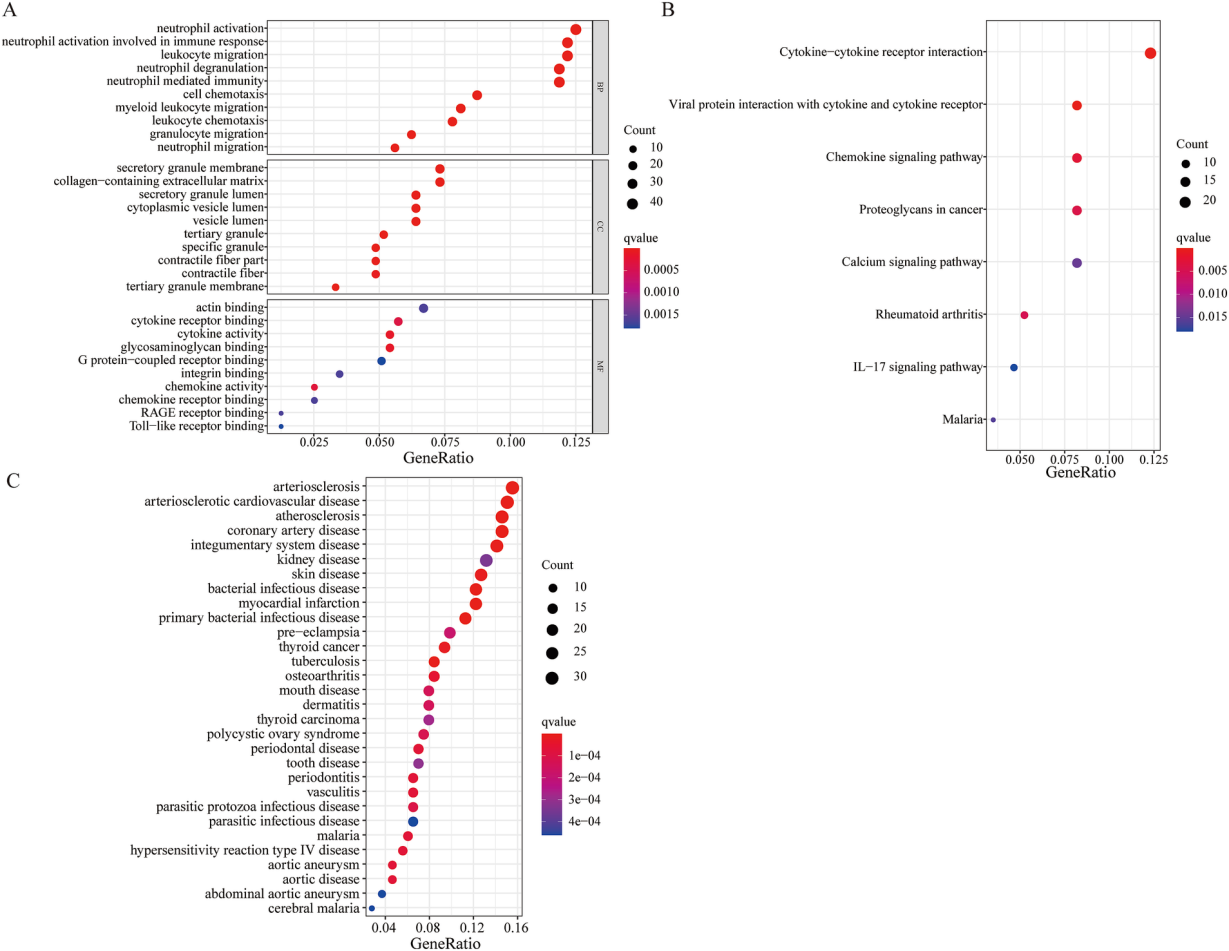
Functional enrichment analysis of the DEGs. **(A)** GO functional enrichment analysis; **(B)** KEGG *pathway* enrichment analysis; **(C)** DO function analysis.

The result of the GO functional analysis indicated that the DEGs were enriched in neutrophil activation, leukocyte migration, neutrophil-mediated immunity, and granulocyte migration (Figure 2A). The KEGG enrichment pathway analysis showed that the DEGs were mainly enriched in the IL-17 signaling pathway, cytokine-cytokine receptor interaction, and the chemokine signaling pathway (Figure 2B; Supplementary Figure 2). The DO functional analysis indicated that the DEGs were related to diseases such as arteriosclerosis, arteriosclerotic cardiovascular disease, and coronary artery disease.

### Screening hub genes using LASSO regression and the SVM-RFE algorithm

**Figure 3:**
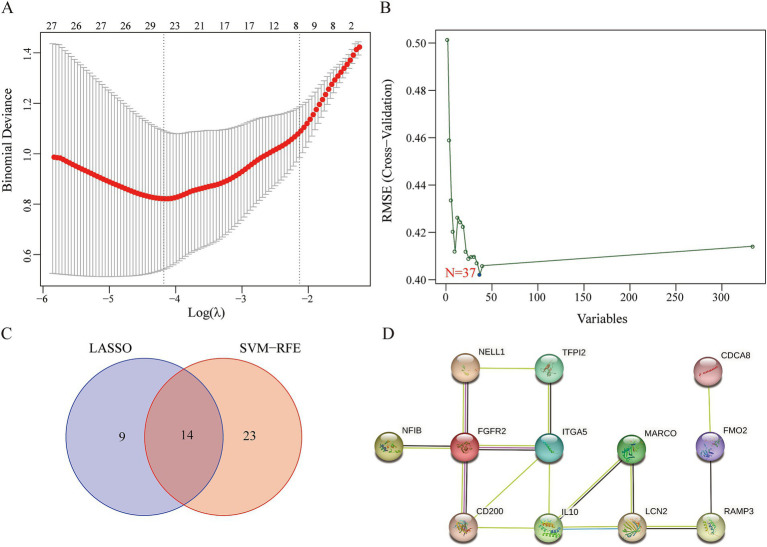
Identification of key genes. **(A)** LASSO regression analysis. **(B)** SVM-REF algorithm. **(C)** The 14 overlapping genes between the LASSO regression and the SVM-RFE algorithm. **(D)** The PPI network of the overlapping genes.

We applied LASSO regression and the SVM-RFE algorithm to filter underlying markers for analyzing hub genes in the PPI network composed of the DEGs. We screened 23 genes using the LASSO regression algorithm (Figures 3A–C). A total of 37 genes were screened using the SVM-RFE algorithm (Figure 3C). Fourteen overlapping genes between the two algorithms were considered hub genes (two genes were excluded because they were unconnected points) (Figure 3D). The AUC value of the fourteen hub genes was over 0.7 (Supplementary Figure 3).

### Identifying key genes

We used CytoNCA, a plugin from Cytoscape, to perform centrality analysis on a PPI network consisting of the 14 central genes. The top 20% of the intersection genes based on degree, betweenness, and eigenvector centrality in the PPI network were considered key genes. The results showed that IL10 and Integrin α5 (ITGA5) were identified as key genes for further analysis (Figure 3D).

### Verifying key genes

**Figure 4:**
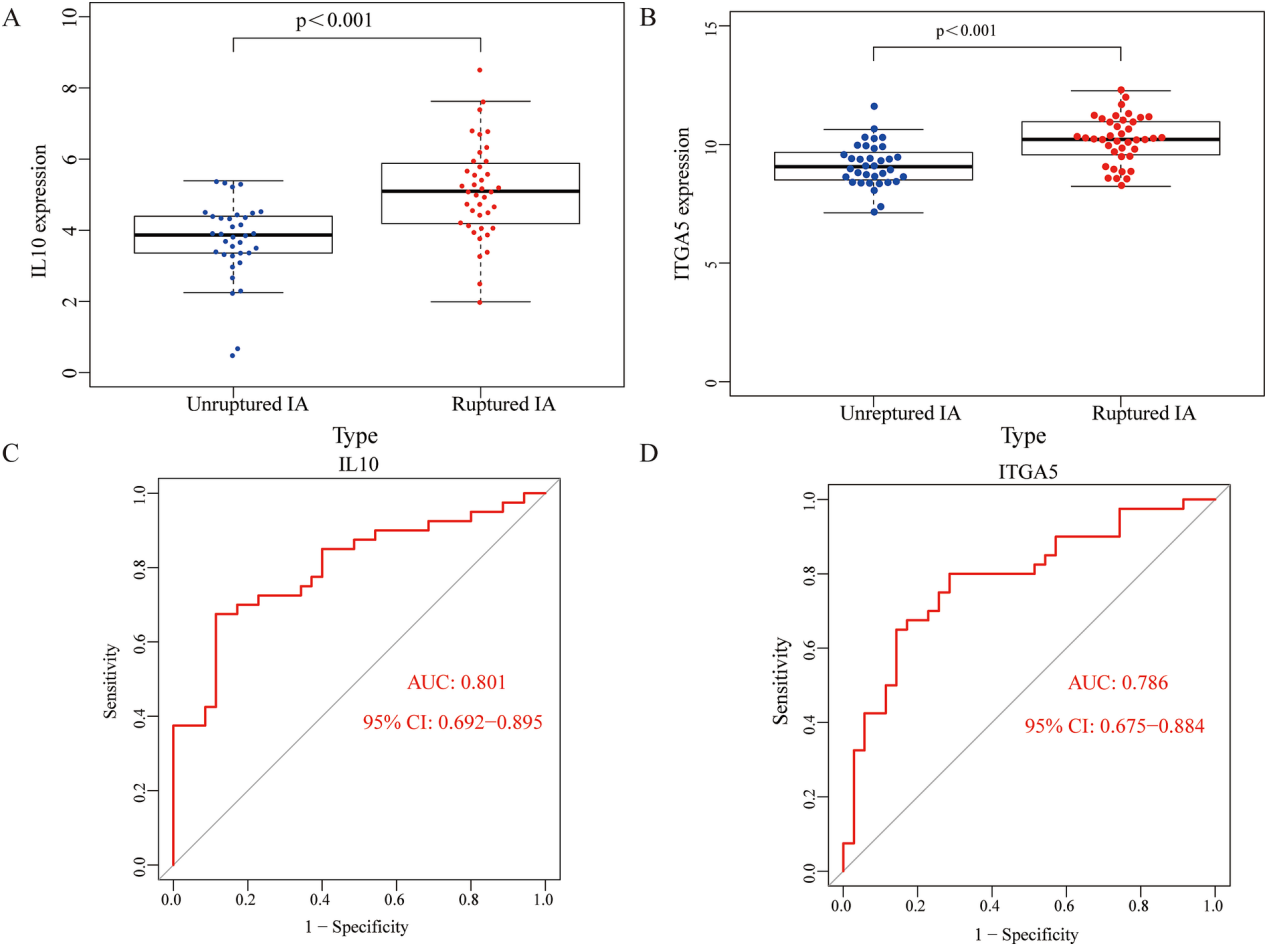
The role of the key genes in the patients with an IA. IL10 and ITGA5 expressions were higher in the ruptured IA group compared to the unruptured IA group **(A,B)**. ROC curves for IL10 and ITGA5 in the ruptured IA group **(C,D)**.

To clarify the role of key genes in patients with an IA, we compared the expression of the key genes in 35 unruptured IA cases and 40 ruptured IA cases. Figures 4A,B and Supplementary Figure 4 show that IL-10 and ITGA5 were higher in the patients with a ruptured IA than in those with an unruptured IA. The AUC values of IL10 and ITGA5 were 0.801 and 0.786, respectively (Figures 4C,D).

### Immune infiltration landscape

**Figure 5:**
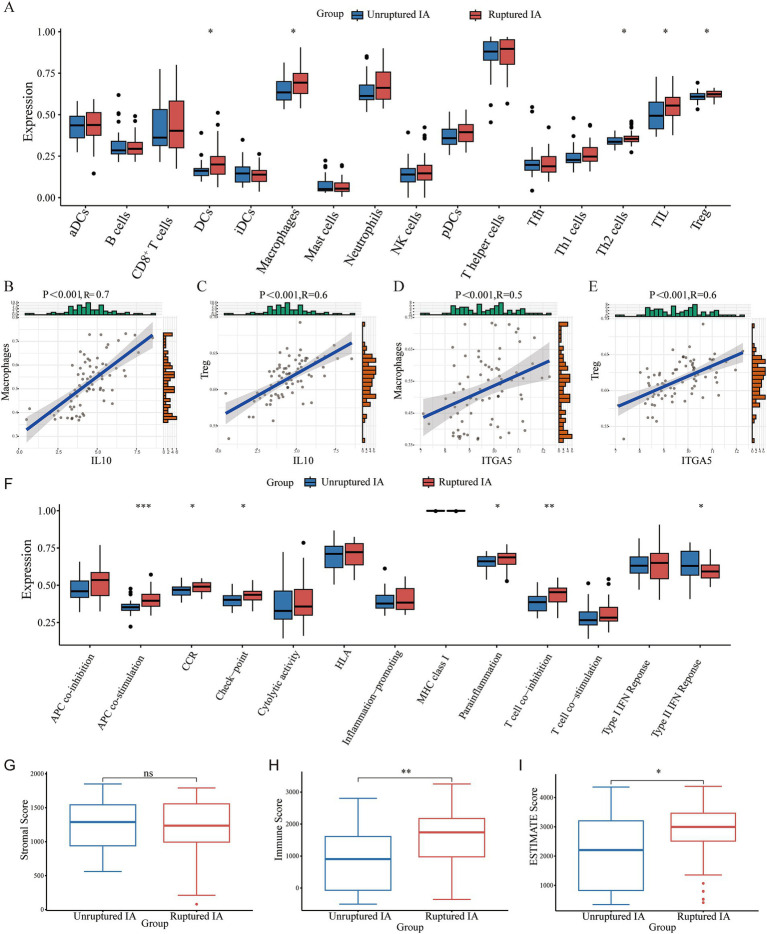
The immune infiltration landscape of the patients with an IA. **(A)** The proportions of immune cells. **(B–E)** The correlation between IL10 and macrophages and Treg cells, respectively. **(F)** Differences in various immune processes between the disease and control groups estimated using the algorithm based on ssGSEA. **(G–I)** The stromal score, immune score, and ESTIMATE score in the ruptured IA and unruptured IA groups.

The enrichment scores of 16 immune cells and 13 immune functions for each sample were quantified using the ssGSEA algorithm. Figure 5A shows the proportions of the immune cells in the IA sample. We then inferred the differences in the proportions of infiltrating immune cells. Compared to the unruptured IA group, we found that the ruptured IA group had relatively higher percentages of dendritic cells (DCs), macrophages, T helper 2 (Th2) cells, tumor-infiltrating lymphocytes (TIL), and regulatory Treg (T) cells (Figure 5A). Previous studies have found that macrophages (20) and Treg cells (21) are associated with whether an IA is ruptured. Therefore, this study further analyzed the correlation between IL10 and these two immune cells, as well as between ITGA5 and the two immune cells. The results showed that the correlation coefficients of IL-10 with macrophages and T cells were 0.7 and 0.6, respectively (Figures 5B,C). The correlation coefficients of ITGA5 with macrophages and T cells were 0.5 and 0.6, respectively (Figures 5D,E).

In addition, APC co-stimulation, CCR, check-point, parainflammation, and T cell co-stimulation were significantly higher in the ruptured IA group than in the unruptured IA group, and the type II IFN response was higher in the unruptured IA group (Figure 5F). The matrix score, immune score, and ESTIMATE score for all samples were estimated using the estimation package. The results showed no significant difference in the stromal score between the ruptured IA and unruptured IA groups. However, the immune score was higher in the ruptured IA group, as was the ESTIMATE score, indicating that the ruptured IA group was in an immune activation state (Figures 5G–I).

## Discussion

An IA is a serious complication and represents one of the most challenging cerebrovascular complications faced by clinical staff (22). Given the complex pathogenesis of IAs, there is an urgent need to identify new biomarkers for early clinical diagnosis and prognosis evaluation, as well as to explore the potential mechanisms of IA development, thereby aiding in the development of treatment strategies (23).

IL-10 is a cytokine with anti-inflammatory and immunomodulatory functions. It inhibits inflammation through various mechanisms. It can downregulate the expression of major histocompatibility complex II (MHC II) on the surface of monocytes, reduce its antigen presentation, downregulate the activity of T lymphocytes, and inhibit the activation, migration, and adhesion of inflammatory cells (24). At the same time, IL-10 can inhibit the synthesis and release of inflammatory factors (25). It also inhibits the cytokine production ability of antigen-presenting cells and the expression of costimulatory molecules (such as CD80 and CD86), thus reducing the inflammatory response (26). It plays a key role in regulating the immune response. It can inhibit IFN-g and IL-2 produced by Th1 cells, as well as IL-4 and IL-5 produced by Th2 cells, thus reducing the activation of immune cells and inflammatory reactions (27, 28). This immunomodulatory effect may help slow down the development of aneurysms. Previous studies have shown an increase in the absolute plasma concentration of IL-10 in the IA lumen (29). Integrins, composed of *α* and *β* subunits, are heterodimeric transmembrane proteins that play the role of a surface adhesion receptor in cell communication. Integrin α5 (ITGA5) participates in the interaction between cells and the extracellular matrix and affects the processes of cell adhesion and proliferation. α5 integrins usually combine with β1 integrins to form α5β1 integrins, which are involved in many biological processes, including cell migration, tissue repair, and tumor invasion (30–32). ITGA5 interacts with various molecules, influencing cell adhesion, migration, and signal transduction. For example, ITGA5 binds to fibroblast activation protein α (FAPα) to form a protein complex that regulates osteoclast differentiation (33). Linli Zheng et al. demonstrated that ITGA5 + synovial fibroblasts may regulate the progression of rheumatoid arthritis (RA) by remodeling proinflammatory microenvironments. Therefore, therapeutic modulation of this subpopulation could be a potential treatment strategy for RA (34).

In our research, the screened 334 DEGs were subjected to functional analysis. The ruptured IAs were mainly enriched in the pathways related to inflammation and the immune response. Based on the LASSO model, SVM-RFE analysis, and the PPI network, two key genes, IL10 and ITGA5, were identified. The accuracy of the key genes screened was further validated using ROC curve analysis. In addition, the key genes showed a significant positive correlation with macrophages and Treg cells based on ssGSEA. The immune score and ESTIMATE score in the ruptured IA group were significantly higher than those in the unruptured IA group.

## Conclusion

In conclusion, we identified two immune-related key genes, IL10 and ITGA5, through a series of screening methods. The ROC curve and immune correlation analysis further confirmed the reliability of our findings. Therefore, IL10 and ITGA5 can serve as potential biomarkers for IA rupture.

## Data Availability

The datasets presented in this study can be found in online repositories. The names of the repository/repositories and accession number(s) can be found at: https://www.ncbi.nlm.nih.gov/gds/?term.

## Supplementary material

SUPPLEMENTARY FIGURE 1 The complex PPI network of the DEGs between ruptured IA and unruptured IA patients.

SUPPLEMENTARY FIGURE 2 The GSEA functional analysis of the DEGs. **(A)** Ruptured IA; **(B)** unruptured IA.

SUPPLEMENTARY FIGURE 3 The ROC curve analysis of the hub genes.

SUPPLEMENTARY FIGURE 4 Analysis on the difference of gene expression between IL10 **(A)** and ITGA5 **(B)**.
