# Supplementary figures and images for: Screening key genes for intracranial aneurysm rupture using LASSO regression and the SVM-RFE algorithm
Source: Front Med (Lausanne). 2025 Jan 6;11:1487224. doi: 10.3389/fmed.2024.1487224 (PMC11743535; doi:10.3389/fmed.2024.1487224)

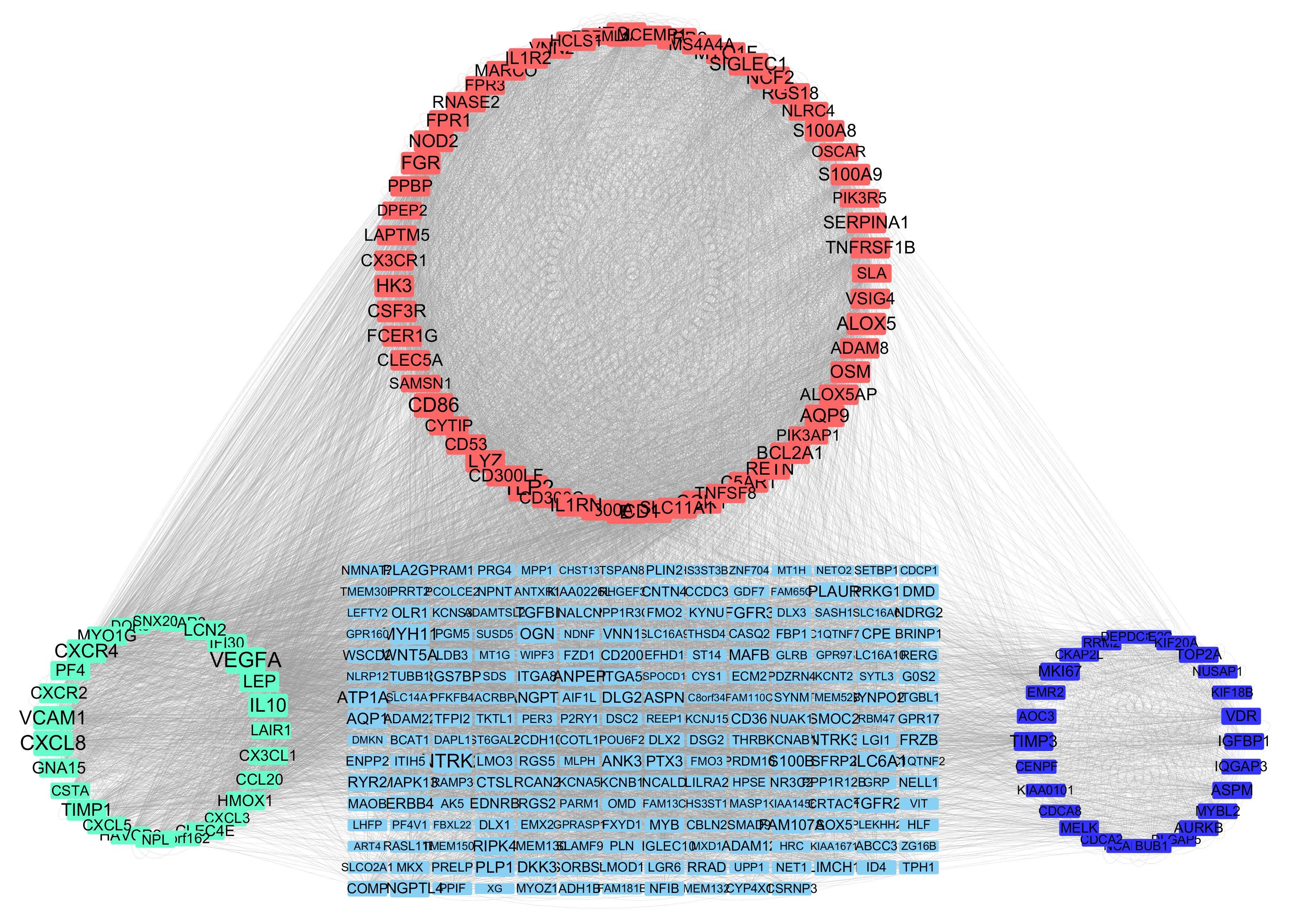

Supplement: SUPPLEMENTARY FIGURE 1 — The complex PPI network of the DEGs between ruptured IA and unruptured IA patients. [file Image_1.JPEG]

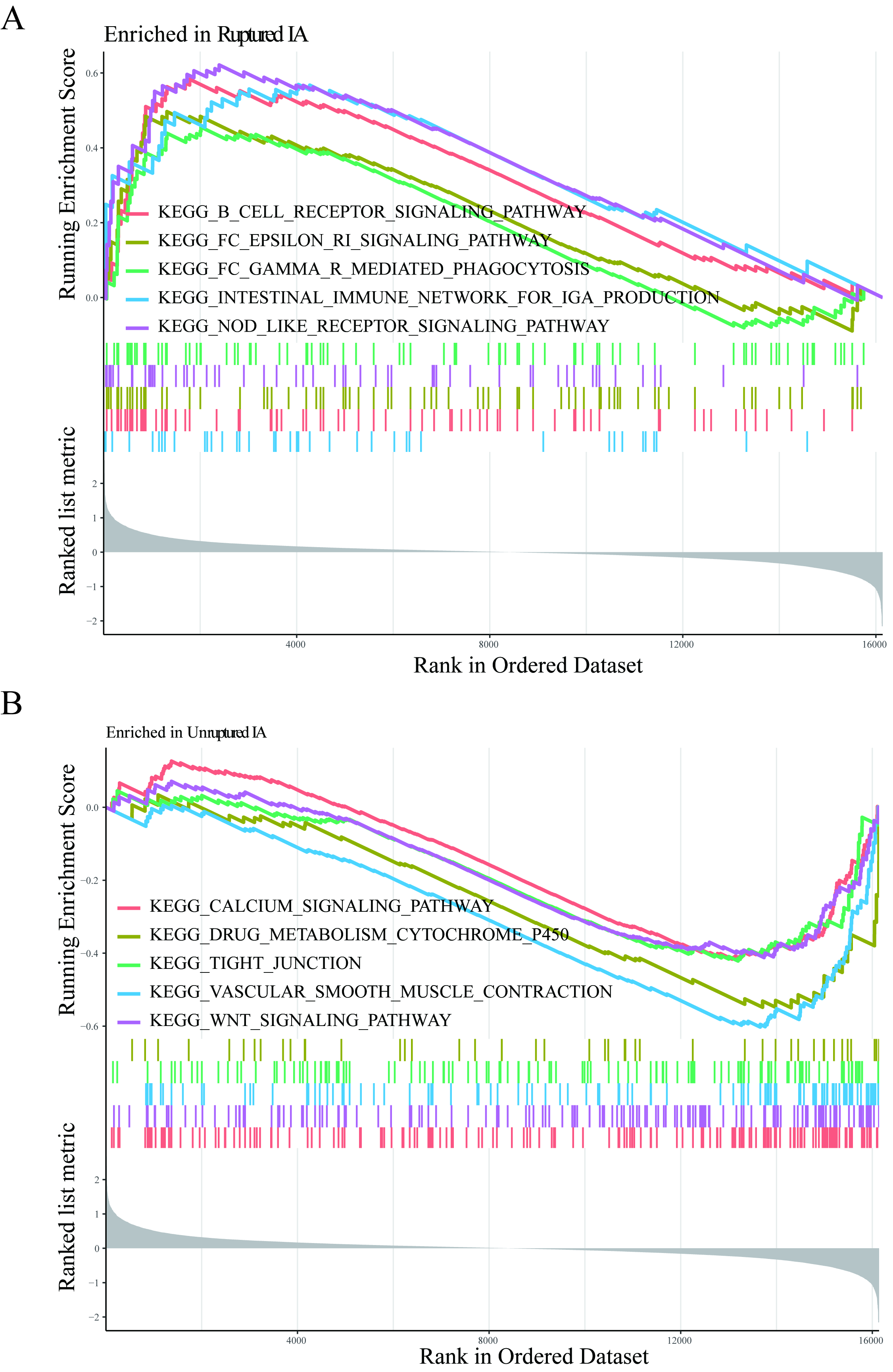

Supplement: SUPPLEMENTARY FIGURE 2 — The GSEA functional analysis of the DEGs. (A) Ruptured IA; (B) unruptured IA. [file Image_2.TIF]

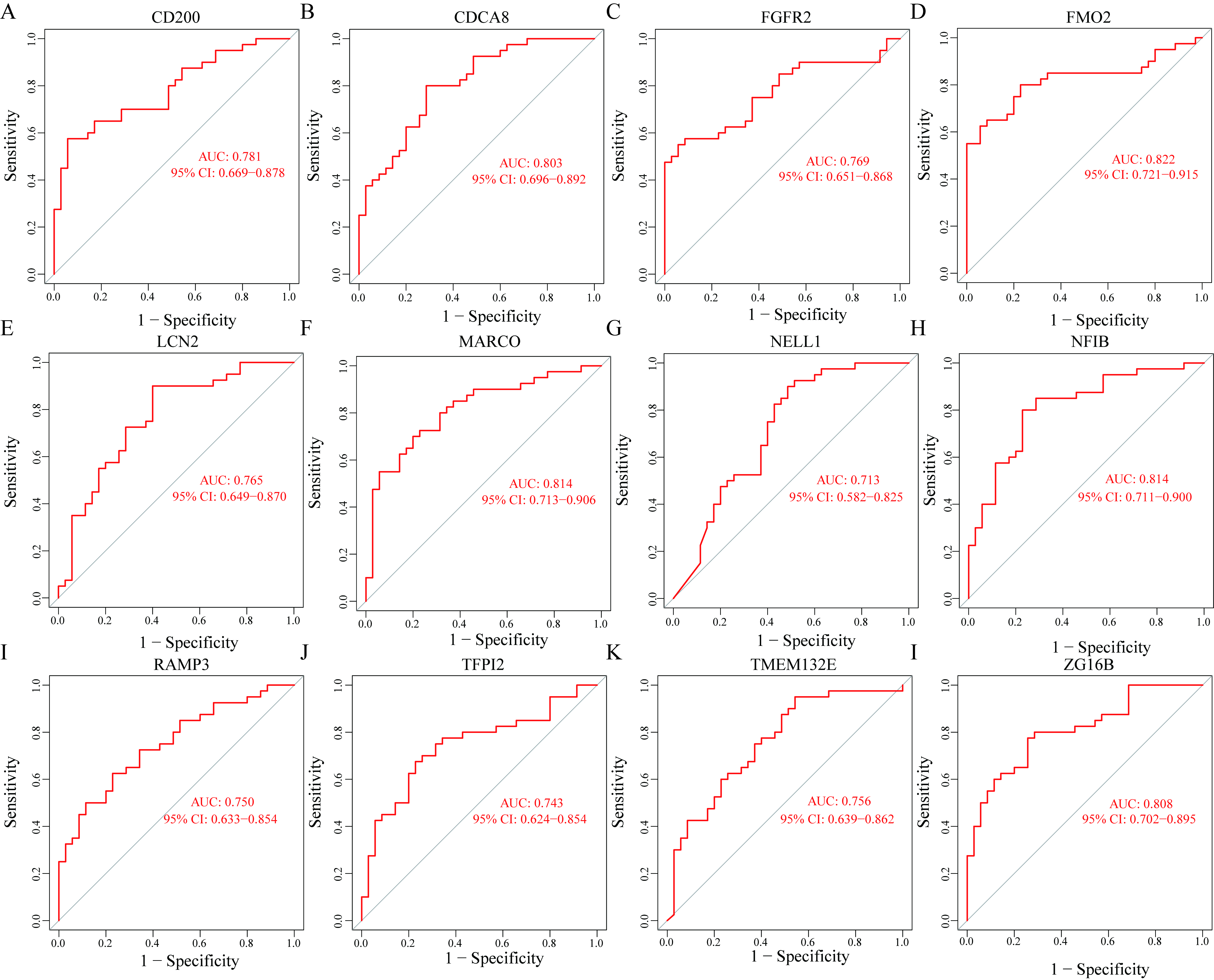

Supplement: SUPPLEMENTARY FIGURE 3 — The ROC curve analysis of the hub genes. [file Image_3.TIF]

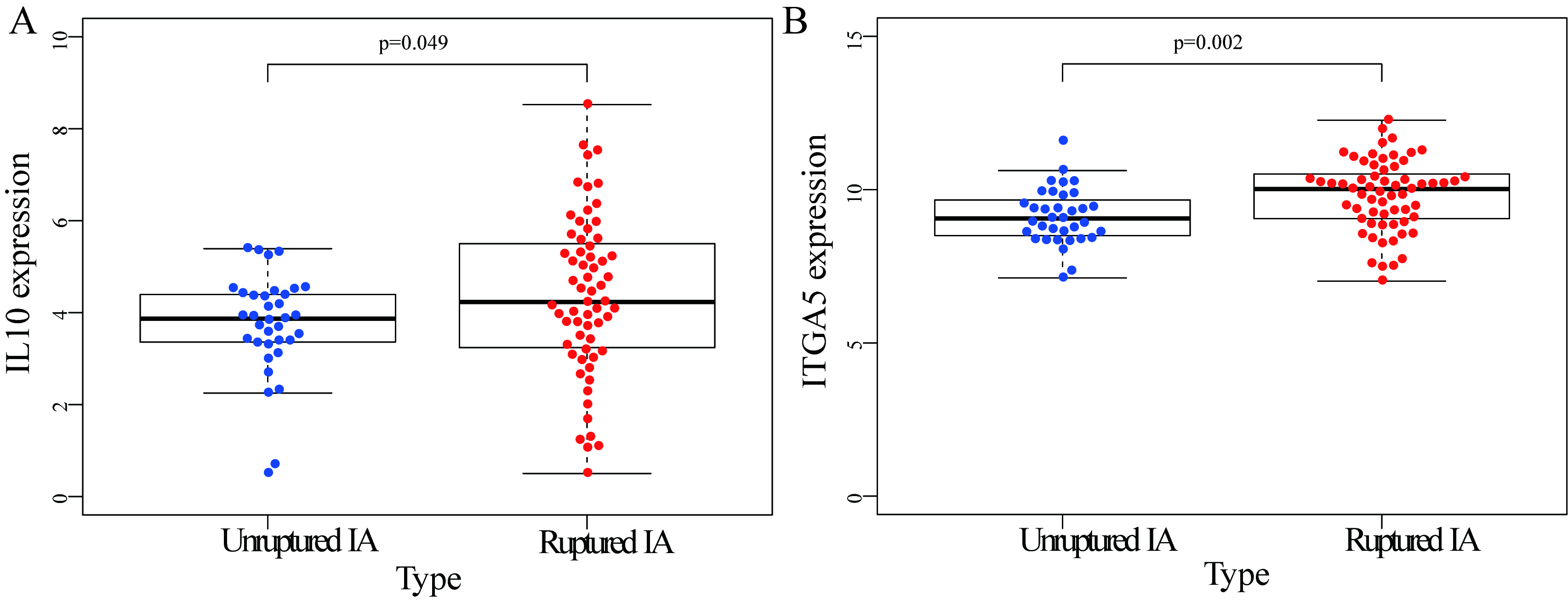

Supplement: SUPPLEMENTARY FIGURE 4 — Analysis on the difference of gene expression between IL10 (A) and ITGA5 (B). [file Image_4.TIF]

Supply evidence of manuscript construction involving different members of the author group.

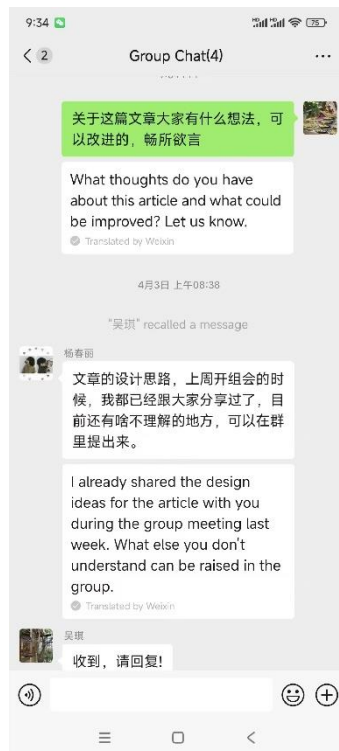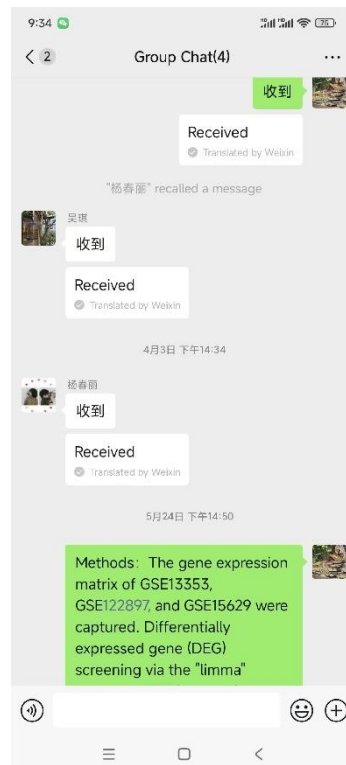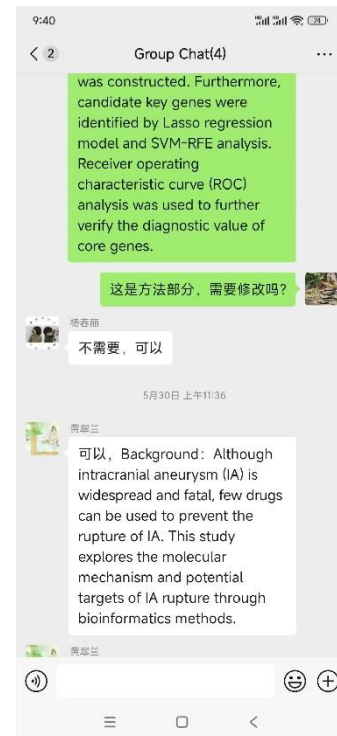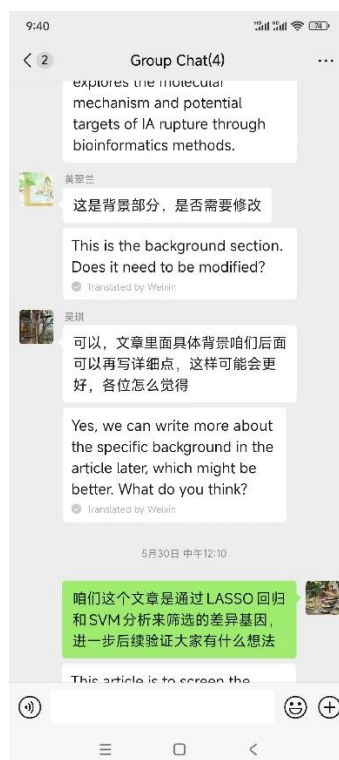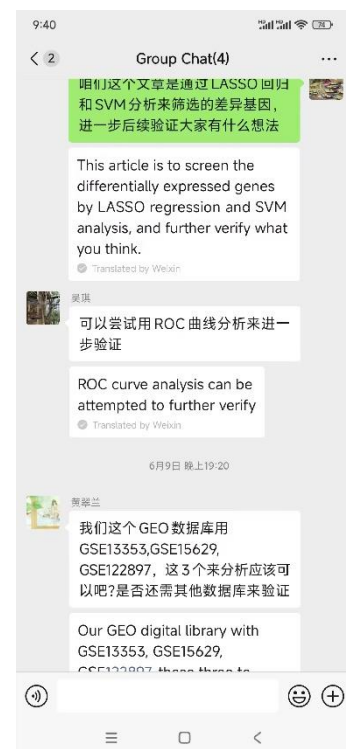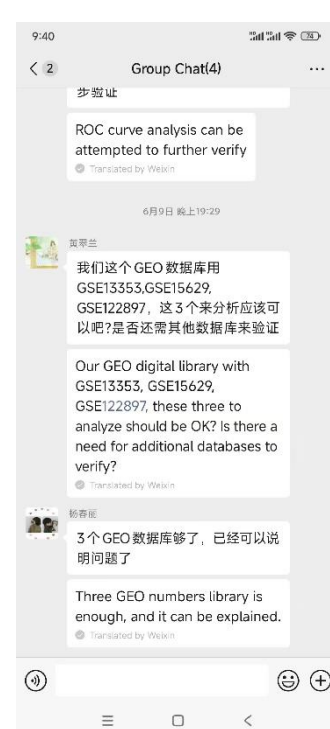

Supplement: Supplementary file 6 [file Data_Sheet_1.PDF]
